# *NOMO-1* gene is deleted in early-onset colorectal cancer

**DOI:** 10.18632/oncotarget.15478

**Published:** 2017-02-18

**Authors:** José Perea, Juan Luis García, Jessica Pérez, Daniel Rueda, María Arriba, Yolanda Rodríguez, Miguel Urioste, Rogelio González-Sarmiento

**Affiliations:** ^1^ Surgery Department, University Hospital 12 de Octubre, Madrid, Spain; ^2^ Digestive Cancer Research Group, 12 de Octubre Research Institute, Madrid, Spain; ^3^ Department of Medicine, Molecular Medicine Unit, Biomedical Research Institute of Salamanca (IBSAL), Institute of Molecular and Cellular Biology of Cancer (IBMCC), University of Salamanca, SACYL, CSIC, Salamanca, Spain; ^4^ Molecular Biology Laboratory, University Hospital 12 de Octubre, Madrid, Spain; ^5^ Pathology Department, University Hospital 12 de Octubre, Madrid, Spain; ^6^ Familial Cancer Clinical Unit, Spanish National Cancer Centre (CNIO), Madrid, Spain; ^7^ Center for Biomedical Network Research on Rare Diseases (CIBERER), Institute of Health Carlos III, Madrid, Spain

**Keywords:** early-onset colorectal cancer, NOMO-1, nodal pathway, array comparative genomic hybridization, 16p13.12-p13.11

## Abstract

To characterize clinical features of a recurrent alteration in 16p13.12-p13.11 in Colorectal Cancer (CRC), mainly in Early-onset subgroup (EOCRC), and to assess the status of *NOMO1*, a gene located in that region, we analyzed differential clinicopathological, familial and molecular features of CRC subsets with and without alterations in the 16p13.12-p13.11, in global and EOCRC groups. We confirmed the region by fluorescence in-situ hybridization, and Quantitative Real-Time PCR analyzed the status of *NOMO1* in different age-of-onset and Microsatellite Instability (MSI)-status CRC subsets. Both age-of-onset subsets were subsequently extended to further confirm *NOMO1* gene changes. 16p13.12-p13.11 alterations were observed in 23.3% of CRCs, and was detected more frequently in EOCRC (33.3%) than in late-onset CRC (16.3%). The group with deletion in 16p showed a higher frequency of females and left-colon locations; a better prognosis; and higher Chromosomal Instability. Within the primary EOCRC population, 34 out of 34 of tumours showed a homozygous deletion in *NOMO1*, while in the late-onset population only 2 of the 17 tumours (11.7%) showed it. In the extended group, we found 61 out of 75 EOCRC patients (81.3%) with homozygous deletion and 7 patients (9.3%) with heterozygous deletion of *NOMO1*; moreover, in the new 50 late-onset patients, the proportions of deletions decreased. Microsatellite-Stable (MSS) EOCRC showed a very high proportion of homozygous loss of *NOMO1* (54 of 59 cases, 91.5%), while the deletion was observed in only 7 out of 16 MSI cases. Deletion of *NOMO1* is a molecular marker predominantly associated with EOCRC, particularly MSS subtypes.

## INTRODUCTION

Early-onset Colorectal Cancer (EOCRC) has an incidence of 2–8% of all Colorectal Cancers (CRCs) and it has increased in the past decades to reach 11% of colon cancers and 18% of rectal cancers [1]. The impact of EOCRC on the population is undeniably important, and until recently the idea prevailed that this subset of CRC occurred mainly in hereditary CRC forms. However, recent studies challenge this statement, since they are primarily Microsatellite Stable (MSS) cases [2–6]. Moreover, EOCRC (except cases with an already known hereditary component) may be a specific subgroup of CRC [3–5], so that a deeper understanding of the underlying molecular mechanisms is essential.

EOCRC has evolved from a controversy on its natural history and prognosis to the characterization of an important heterogeneity within this group [6]. Moreover, it has been proposed that age of onset is a major criterion for subclassifying CRC [3]. Most studies conclude that there are differential features within this age group not only from a clinical point of view but also, and more significantly, according to a number of differential molecular features: a high degree of LINE-1 hypomethylation [7], more frequent chromosomal and genetic alterations (including some susceptibility variants), underlying an inherited or familiar predisposition [8–10], and unique characteristics, including clinical and molecular features and the type of telomere maintenance mechanism [4, 5]. In spite of all these differential molecular features, to date there is no known molecular or genetic target associated with EOCRC.

CRC results from the accumulation of genetic alterations, and somatic copy number alterations (CNAs) play an important role in its development. Genome-wide survey of CNAs provides opportunities for identifying cancer driver genes in an unbiased manner [11]. In an attempt to better characterize EOCRC, we have previously reported results from an array-Comparative Genomic Hybridization (aCGH) study in which we compared early and late-onset CRC [10]. Analysis of the data have revealed a recurrent deletion in chromosome 16p13.12-p13.11, either alone or associated with other changes (Supplementary Figure 1). The aims of our study were to define the possible clinical phenotype of the cases showing this chromosomal alteration as well as to find out genes in this region that could be altered.

## RESULTS

### 16p deletion is more frequent in EOCRC than in late-onset CRC

**Figure 1:**
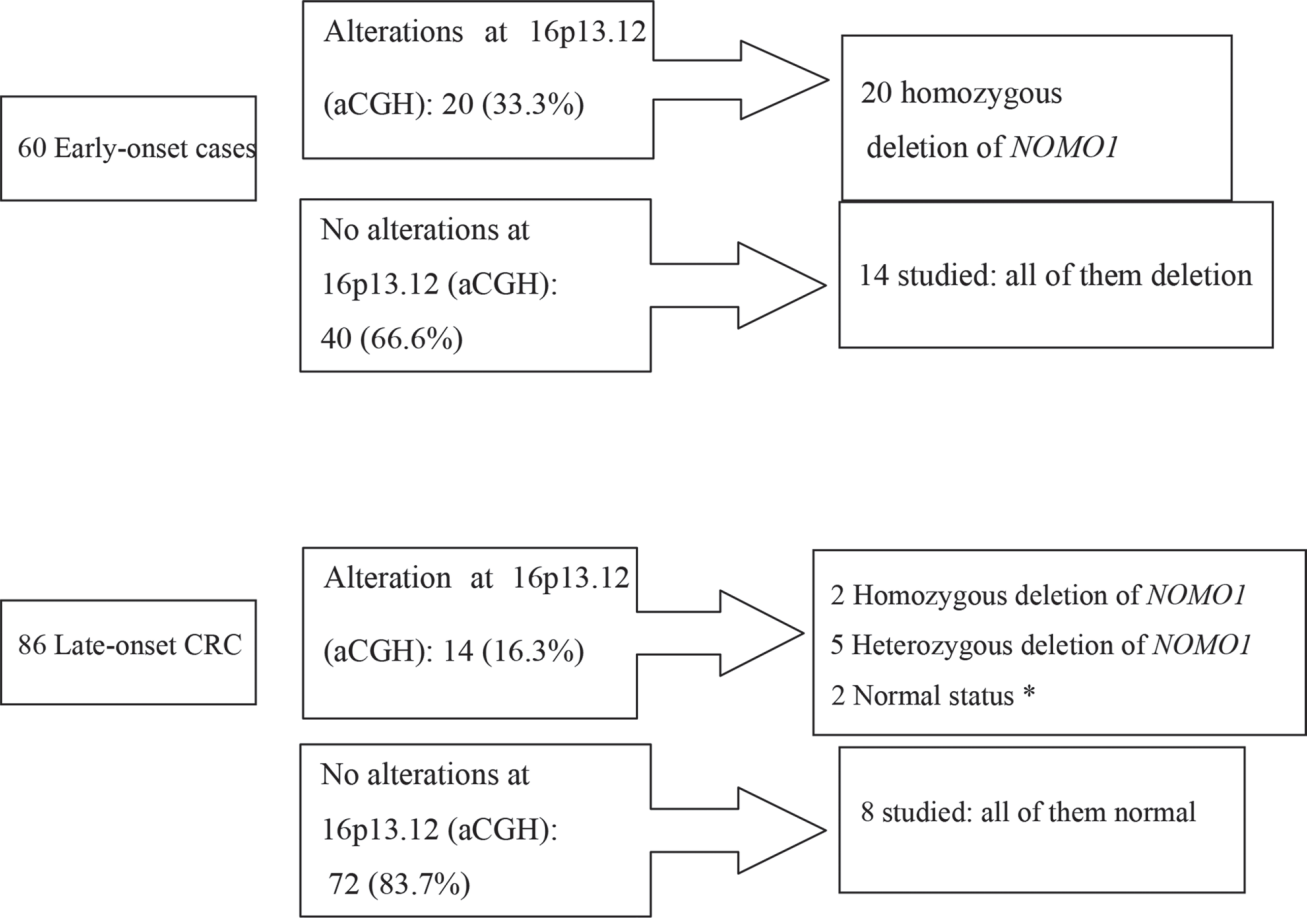
Losses at 16p13.12-p13.11 and *NOMO-1* alterations within the early-onset CRC and late-onset CRC subgroups *Only 9 cases could be analyzed, because of lack of material.

Sixty cases from the EOCRC subset and 86 from the late-onset population were studied by aCGH (the others could not be studied because of lack of material); analysis of the data revealed a recurrent focal deletion in chromosome 16p13.12-p13.11, either as the unique change or associated with other changes (Supplementary Figure 1). This focal alteration was observed in 23.3% (34/146) cases in our series, and was detected more frequently in early-onset CRC (33.3%, 20/60) than in late-onset CRC (16.3%, 14/86) (*p* = 0.028) (Figure 1).

**Figure 2:**
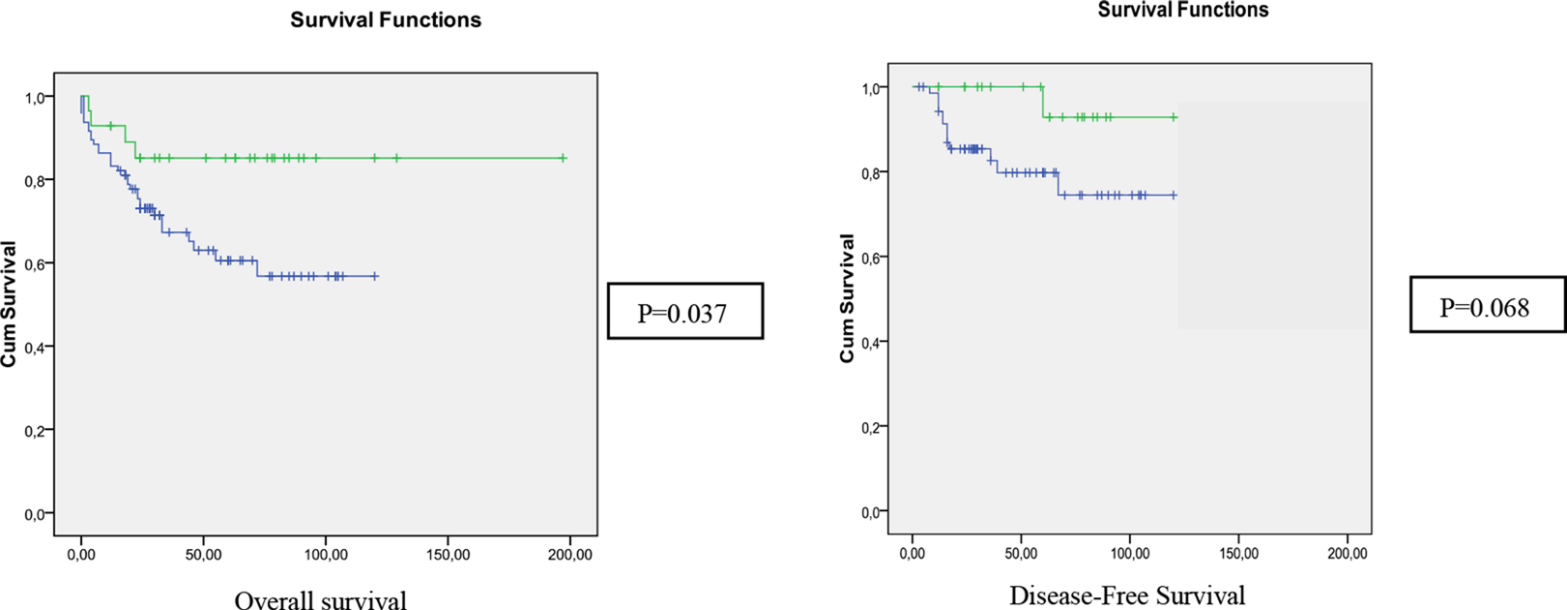
Kaplan-meier curves for overall survival and disease-free survival of the global subset, according to the status of 16p13.12 in aCGH (Green: altered; Blue: normal)

In Table 1 we compare clinico-pathological, familial, molecular and follow-up features of the groups according to 16p status as determined by aCGH. In the global population (without defining any age-of onset criteria), the 16p deletion appeared to be more frequent in females (58.8% vs 39.3%). Moreover, in the group with 16p deletion we observed a very low rate of rectal (17.6% vs 42%) and a high rate of left-colon locations (47.1% vs 27.7%); a better prognosis, and a higher Chromosomal Instability. Kaplan-Meier curves are shown in Figure 2, confirming the better prognosis of the group with 16p13.12-p13.11 deletion, mainly for OS. When we analysed the EOCRC group separately, only tumor location and Chromosomal Instability (number of CNAs) remained statistically significantly different between the patients with and without 16p deletion (Table 1).

**Table 1:** Clinical, pathological and familial features of all CRC cases (global group), and comparison of the subgroups with and without 16p alterations within the global and the early-onset groups

	16p altered Global *n* (%)	16p normal Global *n* (%)	*p* (χ^2^)	16p altered Early-onset *n* (%)	16p normal Early-onset *n* (%)	*p* (χ^2^)
**Patients**	34 (23.3)	112 (76.7)		20 (33.3)	40 (66.7)	
**Mean age of onset (SD)^1^**				38.9 (5.6)	39.4 (4.9)	NS
**Sex**: **Male** **Female**	14 (41.2) 20 (58.8)	68 (60.7) 44 (39.3)	**0.05**	9 (45) 11 (55)	27 (67.5) 13 (32.5)	NS
**Location**: **Right colon** **Left colon** **Rectum**	12 (35.3) 16 (47.1) 6 (17.6)	34 (30.4) 31 (27.7) 47 (42)	**0.024**	7 (35) 13 (65) 0 (0)	6 (15) 15 (37.5) 19 (47.5)	**0.001**
**Tumor differentiation ^2^**: **Poor**	2/30 (6.7)	5/99 (5.1)	NS	2/16 (12.5)	1/33 (3)	NS
**Mucin production ^2^**. **“Signet ring” cells ^2^**.	6/30 (20) 0 (0)	23/99 (23.2) 4/99 (4)	NS NS	5/16 (31.3) 0/16 (0)	9/33 (27.3) 2/33 (6.1)	NS NS
**Modified Astler Coller stage**: **A** **B** **C** **D**	6 (17.6) 18 (52.9) 4 (11.8) 6 (17.6)	13 (11.6) 48 (42.9) 29 (25.9) 22 (19.6)	NS	4 (20) 11 (55) 1 (5) 4 (20)	11 (27.5) 12 (30) 9 (22.5) 8 (20)	NS
**Associated polyps**	25 (73.5)	66 (58.9)	NS	13 (65)	22 (55)	NS
**Mean number of polyps (SD)^1^**	2.3 (2.8)	2.8 (6.3)	NS	2.2 (2.6)	2.8 (8)	NS
**Type**: **Adenomatous** **Hyperplastic** **Mixed**	11 (44) 3 (22) 11 (44)	39 (59) 9 (14) 18 (27)	NS	5 (38.5) 2 (15.4) 6 (46.1)	9 (40.9) 5 (22.7) 8 (36.4)	NS
**Synchronous o metachronous CRCs**.	4 (11.8)	19 (17)	NS	3 (15)	2 (5)	NS
**Recurrence^3^**	2 (7.7)	15 (16.7)	NS	2 (12.5)	5 (15.6)	NS
**Related mortality**	6 (17.6)	36 (32.1)	NS	3 (15)	11 (27.5)	NS
**Disease-free survival (SD)^1^**	55.8 (46.4)	32.9 (31.9)	**0.02**	65.4 (47)	51.7 (36.5)	0.2
**Overall survival (SD)^1^**	61.3 (43.7)	38.7 (30.3)	**0.15**	72 (42.8)	61.1 (29.8)	0.3
**MSI**	4 (11.8)	12 (10.7)	NS	3 (15)	6 (15)	NS
**MMR genes mutations**	3 (8.8)	5 (4.5)	NS	3 (15)	4 (10)	NS
**CIMP-High**	9 (26.5)	22 (19.6)	NS	6 (30)	6 (15)	0.1
**CNA (SD)^1^**	175 (90.5)	110 (75.5)	**0.01**	160 (128.5)	63.7 (67.3)	**0.004**
**Familial history of cancer** **Amsterdam II Positive families**. **Aggregation for Lynch neoplasm**. **Aggregation for Lynch unrelated neoplasm**. **Sporadic cases**.	4 (11.8) 13 (38.2) 8 (23.5) 17 (50)	7 (6.3) 27 (24.1) 22 (19.6) 75 (67)	NS	4 (20) 10 (50) 8 (40) 32 (46,4)	7 (17.5) 19 (47.5) 14 (36) 17 (42.5)	NS

^1^ Statistical analysis was carried out using Student´s *t* test. ^2^ Percentages shown are based on varying total numbers as some cases were excluded because only one biopsy was taken (stage D), or because tumours were severely dysplastic with “*in situ*” carcinoma, and it was not possible to study any other characteristic. ^3^ Cases showing recurrence are those with stage C or less at diagnosis.

SD: Standard Deviation. NS: Not significant. CRC: Colorectal Cancer CIMP: CpG Island Methylator Phenotype. MSI: Microsatellite instability. MMR: Mismatch Repair system. CNA: Copy Number Alterations per case.

### 16p13.12-p13.11 deletion causes loss of *NOMO1* gene

Analysis of 16p13.12-p13.11 region by Fluorescence in-situ hybridization analysis (FISH) showed a minimal common region between bases 14738223 and 15353060 with a size of 614838 bp (hg19) (http://genome.ucsc.edu). This region contains the genes *BFAR* (bifunctional apoptosis regulator), *PLA2G10* (phospholipase A2), *NPIPA2* (nuclear pore complex interacting protein) *ABCC6P2* (ATP-binding cassette, sub-family C, member 6 pseudogene 2), *NOMO1* (NODAL modulator 1), *PDXDC1* (pyridoxal-dependent decarboxylase domain containing 1), *NTAN1* (N-terminal asparagine amidase), *RRN3* (RRN3 RNA polymerase I transcription factor homolog), and *PKD1P6* (polycystic kidney disease 1 (autosomal dominant) pseudogene 6).

We analysed the status of the *NOMO1* gene in both age-at-onset subgroups. Within EOCRC, all 20 cases with a cytogenetic 16p13.12-p13.11 deletion showed homozygous deletion of *NOMO1*. Unexpectedly, we also observed 14 cases without cytogenetic evidence of a 16p13.12-p13.11 deletion that presented also homozygous deletion of *NOMO1* (Figure 1). Thus, within the EOCRC population, 34 of 34 studied tumors showed a homozygous deletion in *NOMO1* (100%). Lack of material prevented us to further analyse the remaining cases.

Within late-onset CRC, we were able to study 9 of the 14 cases showing cytogenetic 16p13.12-p13.11 deletion: only two of these cases showed homozygous loss of *NOMO1*, five showed a heterozygous deletion and the other two showed *NOMO1* wild-type (Figure 1). We also studied 8 late-onset CRC without 16p deletion, and all of them had *NOMO1* wild-type (Figure 1). In summary, only 2 out of 17 late-onset CRCs (11.7%) showed a homozygous deletion in *NOMO1*.

To further investigate the high rate of *NOMO1* loss observed in the preliminary series of EOCRC (34 out of 34), we increased the number to 75 early-onset cases as mentioned in the Methods section. Results are shown in Table 2. Of the 41 new cases, 27 showed *NOMO1* homozygous deletion, 7 showed heterozygous deletion, and 7 showed *NOMO1* wild-type. Taken together, from a total of 75 EOCRC cases we found 61 patients (81.3%) with a homozygous loss; 7 patients (9.3%) with a heterozygous loss; and 7 patients (9.3%) without loss of *NOMO1*. Of the other 50 late-onset CRC added, only one showed *NOMO1* homozygous deletion, 4 showed heterozygous deletion, and 45 showed *NOMO1* wild-type. Taken together, from a total of 67 late-onset cases, we found 3 patients (4.5%) with a homozygous loss; 9 patients (13.4%) with a heterozygous loss; and the rest without loss of *NOMO1*.

**Table 2:** Alterations in *NOMO1* in early-onset CRC in the primary group, the subset extension group, and the global CRC group

	Primary group		Subset extension group		GLOBAL	
	MSS*n* (%)	MSI*n* (%)	MSS*n* (%)	MSI*n* (%)	MSS*n* (%)	MSI*n* (%)
***NOMO1*Homozygous deletion**	31 (100)	3 (100)	23 (82)	4 (31)	54 (92)	7 (44)
***NOMO1*Heterozygous deletion**	0	0	2 (7)	5 (38)	2 (3)	5 (31)
***NOMO1* Normal**	0	0	3 (11)	4 (31)	3 (5)	4 (25)

Primary group: Preliminary series of early-onset CRC, from the aCGH study. Subset extension group: Expansion of the series with Early-onset CRC patients from three different institutions, in order to confirm the preliminary results. GLOBAL: The sum of the two previous. Differences between MSI groups according to the *NOMO1* status in the Global group: *p* < 0.001.

MSI: Microsatellite Instability. MSS: Microsatellite Stability. Ratios are calculated for subgroups of MSI-status.

We divided our EOCRC cases according to Microsatellite Instability (MSI) phenotype. MSS-EOCRC showed a very high proportion of homozygous *NOMO1* deletion (54 of 59, 91.5%), while it was present only in 7 of 16 MSI-EOCRC cases (43.74%). Heterozygous deletion of *NOMO1* appears to be rare in MSS-EOCRC (3.3%, 2/59) and relatively common in MSI-EOCRC cases (31.2%, 5/16) (Differences between MSI groups according to the *NOMO1* status in the Global group: *p* < 0.001). Fourteen out of the 16 MSI cases showed germline mutations in the Mismatch Repair (MMR) genes, so that they were defined as Lynch syndrome cases. Only one of the others showed *MLH1* gene promoter hypermethylation. These last two, without MMR germline mutation, exhibited homozygous *NOMO1* deletion, showing the Lynch syndrome cases all the three possible *NOMO1* mutation status.

### NOMO1 deletion is somatic

We analysed *NOMO1* in DNA obtained from peripheral blood samples of 13 individuals with EOCRC and homozygous deletion in their tumours. None of these cases showed *NOMO1* deletion in their peripheral blood, which strongly suggests that *NOMO1* deletion is somatic.

## DISCUSSION

Deletions of 16p13 observed by aCGH have been associated with multiple congenital anomalies [12]. In cancer, these changes were previously reported in Perivascular Epithelioid Cell tumors (PEComa) and prostate cancer [13, 14]. A small deletion in chromosome 16p13.2 affecting the alternative splicing factor *RBFOX1* was found at a significantly higher rate in the CRC British Bangladeshi patients (50%) than in CRC British Caucasians patients (15%), with the Bangladeshi CRC patients being considerably younger [15]. However, this gene is located at a more telomeric position than the deletion we have pointed out, and we therefore decided to further study the involvement of the 16p deletion in our series of colorectal tumors. Interestingly, the 16p13.12-p13.11 region we observed was significantly more frequently deleted in EOCRC than in late-onset CRC and showed some important clinical and prognostic implications. Apart from a better prognosis, we observed the almost complete absence of rectal tumors within the EOCRC subset with 16p deletion. The predominance of left-colon cancer and a high chromosomal instability in these cases are also remarkable. Different nearby regions have been related with prognostic aspects. For example, losses at the 16p13.3 region have been described to lead to poor prognosis in CRC; this region bears a total of 167 known genes among which the tumor suppressor gene *AXIN1* [11, 16]. The apparent contradiction between better prognosis and high chromosomal instability of cases with 16p deletion observed in the present study should be explained by of the greater proportion of Microsatellite And Chromosomal Stable tumours within EOCRC, which are the ones showing a worse prognosis in this particular subset of CRC.

One of the genes located in 16p13.12-p13.11 is *NOMO1*. Unexpectedly, we found *NOMO1* deletion not only in cases with cytogenetic deletion in 16p, mainly in the EOCRC, but also in most early-onset cases without 16p deletion (34 out of 34 EOCRC cases studied by aCGH and FISH), while it barely reached 12% in the late-onset CRC subset. The identification of deletions of *NOMO1* gene detected by qRT-PCR highlight the role of this gene in colorectal carcinogenesis, especially in early-onset tumors. However, to date there is no evidence that *NOMO1* is related with carcinogenesis. Its main function currently known is to form part of a protein complex that antagonizes Nodal signaling, a pathway essential for patterning of the early embryo during mesoderm and endoderm induction as well as for the specification of left–right asymmetry [17]. According to the Cancer Gnome Atlas data alterations involving 16p13 appears to have a limited role in CRC that is confirmed in our analysis of other tumours [18].

When we extended the analysis, we confirmed the important proportion or EOCRC cases showing *NOMO1* loss, and preliminary findings also indicate that this loss is specific of CRC and, more significantly, that it appears to be an important clinical marker of MSS EOCRC as we observed the deletion in more than 90% of the cases. Its possible carcinogenetic role remains uncertain but there is recent evidence supporting the possibility that *NOMO1* could act as a tumor suppressor gene: Nodal pathway activity is upregulated in human cancers such as malignant melanoma [19]; the upregulation of Cripto-1, a protein involved downstream of Nodal signaling is observed in many epithelial cancers like CRC [20], and Cripto-1 overexpression promotes tumorigenesis in xenografts and transgenic mice [21]. Moreover, collectively, Nodal signaling pathway promotes the self-renewal of human Colon Cancer Stem cells and mediates carcinogenesis of human CRC in an autocrine manner through Smad2/3 pathway [22]. As *Nomo1* antagonizes the Nodal signaling pathway [23], the deletion of *NOMO1* could lead in a downregulation of the protein and consequently, an upregulation of the Nodal signaling pathway.

In summary, apart from the clinical value of loss of 16p13.12-p13.11, we identify loss of *NOMO1* as a molecular marker mainly associated with EOCRC, and particularly with MSS subtypes. Our findings may serve as a starting point for further studies to confirm the potential carcinogenetic value of this deletion, which would place *NOMO1* in a suitable position as a potential therapeutic target for EOCRC treatment.

## MATERIALS AND METHODS

### Families, samples and data collection

A total of 82 consecutive individuals with CRC diagnosed at an age of 45 years or younger were collected from our institution. We also collected 97 consecutive individuals who were diagnosed during the same period with CRC but at an age of 70 years or older, to compare with the EOCRC group. These groups have been described previously [4, 10]. We collected clinicopathological data and analyzed MSI status, the mutational state of MMR genes, and the CpG methylation phenotype of all cases. Follow-up was at least 5 years from surgery, and Disease-Free Survival (DFS) and Overall Survival (OS), recurrence and cancer-related death for each case were determined. Details of these studies have been previously reported [4, 10].

In order to confirm our findings, we expanded our series with EOCRC patients from three different institutions (University Hospital of Salamanca, Familial Cancer Clinical Unit of the Spanish National Cancer Research Centre, and 12 de Octubre University Hospital) obtaining 41 additional cases. The main clinical features of these patients were also recorded. We collected as well another 50 late-onset CRC from University Hospital of Salamanca, all of them showing MSS.

### Chromosomal instability: array comparative genomic hybridization (aCGH)

Sixty early-onset CRC cases and 86 late-onset CRCs were studied by array-CGH using oligonucleotide microarrays (Roche NimbelGen, Inc., Reykjavik, Iceland) in order to indentify CNAs as previously reported (10). Each genomic region exhibiting a copy number change was examined using the UCSC genome browser (http://genome.ucsc.edu/) to determine the location and significance of the change.

### Fluorescence in-situ hybridization analysis (FISH)

To confirm the gains and losses detected by array-CGH, FISH analysis was performed using BAC clones 354N7 mapped to 16q22.1 (bases 68,727,161-68,887,391) and CTD 2504F3 mapped to 16p13.1 (bases 15,982,491-16,190,907), as previously described (NCBI16/hg18) [24]. These clones were selected from the same BAC clone library used for the BAC-array studies (Wellcome Trust Sanger Institute, Cambridge, UK). DNA from the BAC clones was isolated and directly labelled with either Spectrum Green-dUTP or Spectrum Orange-dUTP (Vysis, Downers Grove, IL), by nick translation and hybridized as previously described [24]. All BAC clones were first hybridized to normal human metaphase chromosomes in order to verify their location.

Paraffin-embedded tissue sections (4-μm thick) were deparaffinized, dehydrated and air-dried. The slides were placed in 2 mM EDTA (pH 9) for 15 minutes. After cooling, the slides were transferred to a Coplin jar containing 40 ml of 0.9% NaCl (pH 1.5) and 160 mg of pepsin (Sigma) preheated to 37°C. Following incubation for 15 minutes, the sections were dehydrated in an alcohol series and fixed in 3:1 methanol:acetic acid for 10 minutes. The slides were then incubated in a Hybrite hybridization chamber (Vysis) for 12 minutes at 72°C, followed by 15 to 20 hours at 37°C. After hybridization, the slides were washed in standard solutions of saline citrate. Nuclei were counterstained with DAPI (Vector Laboratories Inc.). The images were captured with an Olympus BX60 epifluorescence microscope coupled to a CCD camera and evaluated with Cytovision software (Applied Imaging). Approximately 400 non-overlapping tumor cells were evaluated (Supplementary Figure 2).

### Quantitative real-time PCR

For real-time quantification of target gene expression, one-step real-time Polymerase Chain Reaction (RT-PCR) was performed using FastStart Universal SYBR Green Master (ROX) in a StepOnePlus^™^ Real-Time PCR System (Life Technologies-Invitrogen, California, U.S.A.). A fragment of the *NOMO1* gene was amplified from the DNA of patients and controls using the following primers: F: 5′-agctccatgtggatggagtc-3′ and R: 5`-acggatgaagtacagagttc-3. As internal control, the 36b4 gene was amplified from the same DNA using the primers: F: 5′-cagcaagtgggaaggtgtaatcc-3′ and R: 5′-cccattctatcatcaacgggtacaa-3.

Ten μl RT-PCR of a mix containing 15 ng of total DNA, 1 μl of the primer dilution, 4 μl FastStart Universal SYBR Green Master and 4 μl H_2_O were used for amplification. One-step RT-PCR reactions were carried out in 96-well optical reaction plates, covered with MicroAmp^®^ Optical Adhesive Film (Life Technologies-Invitrogen, California, U.S.A). Cycling was as follows: 10 minutes at 95°C followed by 40 cycles of 95°C for 15 seconds, 58°C for 45 seconds and 72°C for 15 seconds. RQ Manager software was used to analyse the values.

The comparative Ct method (2^−ΔΔCt^) was used to calculate the relative expression levels of each amplicon. RT-PCR specificity of each PCR reaction was verified by melting curve analysis.

### Statistical analyses

Continuous variables were expressed as mean values plus/minus standard deviation (SD), and categorical variables were expressed as number of cases and their percentage. Differences were considered significant when *p* < 0.05. For associations between discrete variables, statistical analyses were performed using Pearson's Chi Square (χ^2^) Test for parametric variables, and Fisher's Exact Test for non-parametric variables. For continuous variables, Student's *t* test was used. The SPSS v.11.5 for Windows (SPSS, Inc., Chicago, IL) statistical package was used. The Kaplan–Meier method was used to describe the distribution of survival time, and the log-rank test was applied. For analyses of colorectal cancer-specific mortality, death as a result of CRC was the primary end point, and deaths from other causes were censored. The same analysis was carried out for disease-free survival time, using recurrence as the primary endpoint. For aCGH analysis, statistics were as published before [10].

## SUPPLEMENTARY MATERIALS AND METHODS AND FIGURES



## Abbreviations

aCGH: array-Comparative Genomic Hybridization
CNAs: Copy Number Alterations
CRC: Colorectal Cancer
DFS: Disease-Free Survival
EOCRC: Early-onset Colorectal Cancer
FISH: Fluorescence in-situ hybridization
MMR genes: Mismatch Repair genes
MSI: Microsatellite Instability
MSS: Microsatellite Stability- Microsatellite Stable
PCR: Polymerase Chain Reaction
SD: Standard Deviation.
