# Supplementary material for: NOMO-1 gene is deleted in early-onset colorectal cancer
Source: Oncotarget. 2017 Feb 18;8(15):24429–36. doi: 10.18632/oncotarget.15478 (PMC5421859; doi:10.18632/oncotarget.15478)
Supplement: Supplementary file 1 [file oncotarget-08-24429-s001.pdf]

NOMO-1 gene is deleted in early-onset colorectal cancer

Supplementary Materials

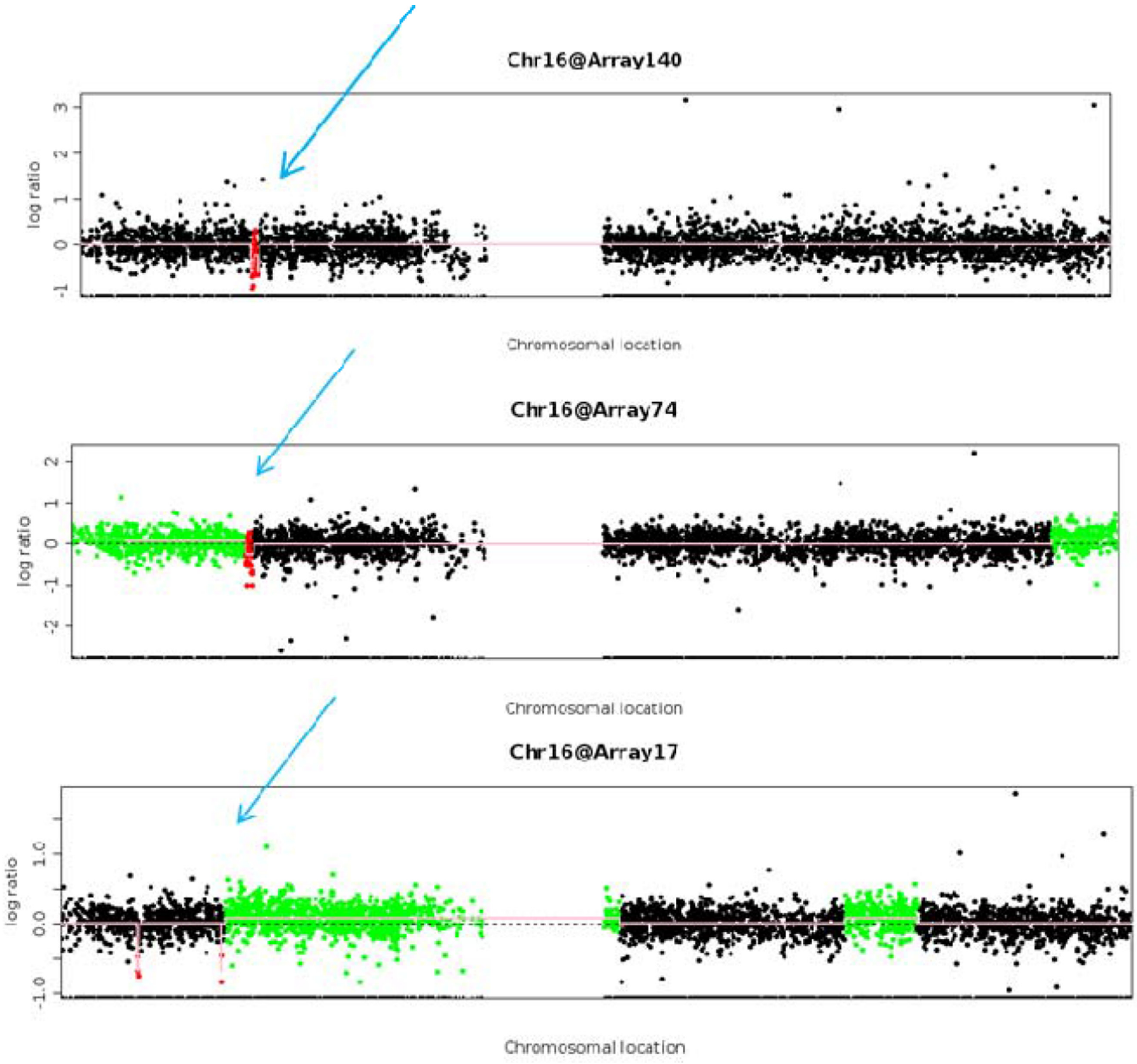

Supplementary Figure 1: Results of segmentation and aberration calling for chromosome 16 of three samples. Analysis of this chromosome shows a short deletion at the p-arm. Normalized log-ratios of probe intensities are plotted as dots of different color. Gained copy number are shown in green, deletions are shown in red and normal copy numbers are shown in black. The segmentation result is shown by the pink lines which correspond to the means of log-ratios of the segment.

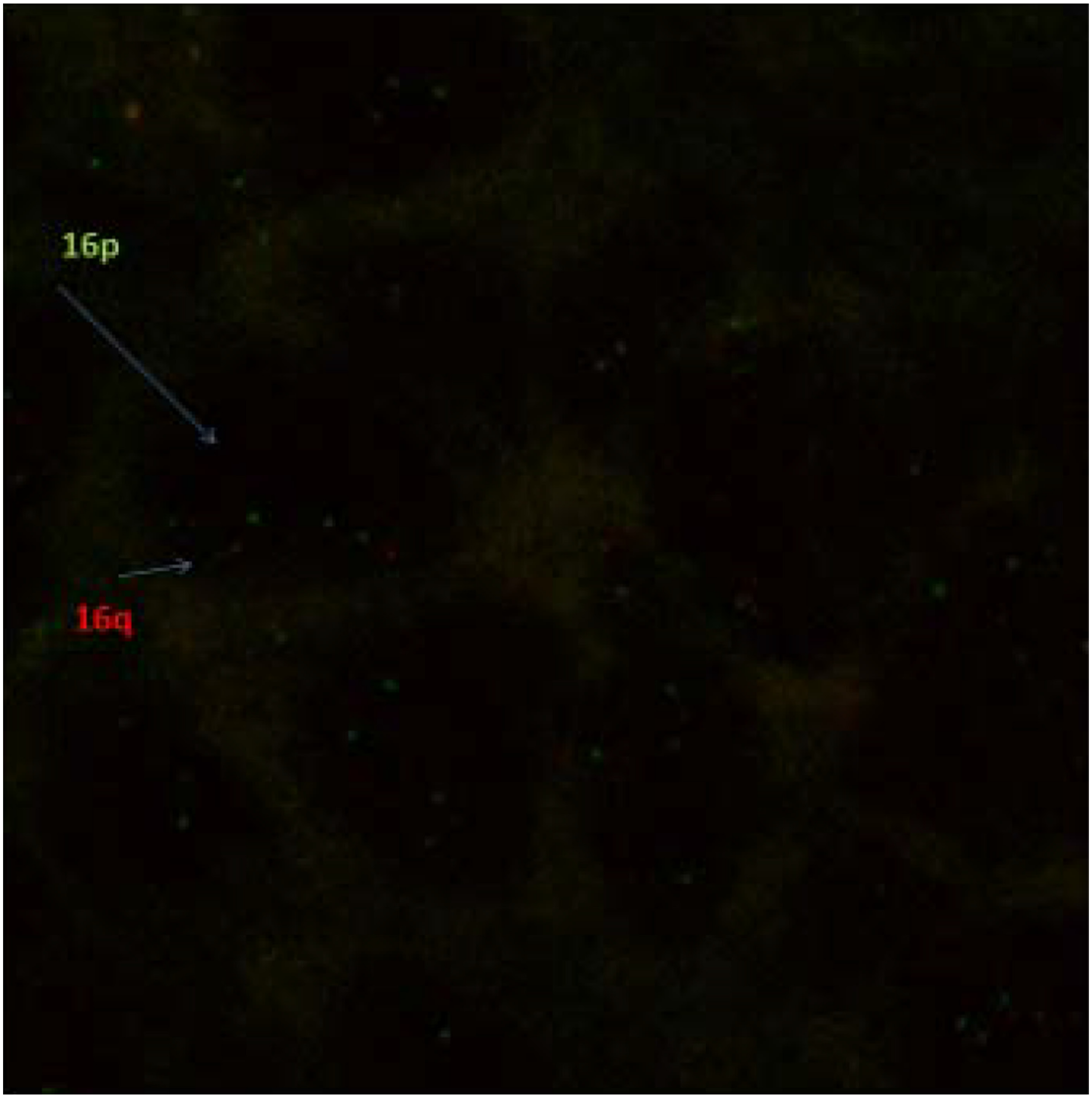

Supplementary Figure 2: Confirmation of cases with aCGH alterations in 16p13 at the same point of rupture. Fluorescence *in situ* hybridization was carried out with the 354N7 probe (16q) (red color), and with the 2504F3 probe (16p) (Green color) (16:16043434-16236931), which encodes the *ABCC1* gene (ATP-binding cassette, sub-family C (CFTR/MRP), member 1).

SUPPLEMENTARY MATERIALS AND METHODS

Interpretation of the qRT-PCR

To interpret the qRT-PCR, the comparative Ct method ($2^{-\Delta\Delta Ct}$) was used to calculate the relative expression levels of each amplicon. When the data is normalized, the value 1 is assigned to the DNA control. Samples with a value around 0.5 show loss of the gene in one of the alleles (heterozygous deletion). Samples with a value around 0 show homozygous loss of the gene.

To validate the technique we use the following controls

As internal control, we have amplified a fragment of a single copy gene: part of exon 1 of *LEMD3* gene using the following primers: LEMD3 F: 5′-GCGGCTGCCGGGAGTCTAGACAG-3′; R:5′-GGTAGG TATGATTGGAGCCCG-3′. -A RBFOX1 gene fragment (part of exon 2) that is located in a chromosomal region close to these three genes (F:5′-TCTGCATGGTGGCTCCTCA T-3′ R:5′- AAGGCTGAGCCATTGTGTCA-3′).

Two differents fragments of the *PKD1* gene: the exon 13 of this gene that has a pseudogen, this fragment has two copies in each allele (F:5′- GTGGAGGG AGGGACGCCAA-3′ R:5′- ACAGGGAAACCGAGGC TCAGAAA-3′). And the exon 30, a fragment of a single copy gene (F:5′- CTACAGGTGGGTGCCGTAGG-3′ R:5′-CGCCTTTCCCTCTGGCTGC-3′).

In addition, we also amplified the exon 5 of the STS gene (F:5′-TCCTTTACAGGAAGATGAAG-3′ R:5′-CATTACCAACCTGATAGTTTT-3′). This gene is located on the "X" chromosome therefore, in women there is a copy of this gene in each allele whereas in men there is only a copy in one of the alleles.

To analyze the samples we used as control gene *LEMD3* and as DNA controls we used DNA extracted from samples of different tissues in paraffin blocks and samples of DNA extracted from peripheral blood.
